# A new 222-m diameter lunar crater

**DOI:** 10.1126/sciadv.aeh7812

**Published:** 2026-09-16

**Authors:** Mark S. Robinson, Aaron K. Boyd, Prasun Mahanti, Madeleine R. Manheim, Emerson J. Speyerer, Julie D. Stopar, Robert V. Wagner

**Affiliations:** ^1^ Intuitive Machines, 101 E. Jackson St., Phoenix, AZ 85004, USA.; ^2^ Lunar and Planetary Institute, Universities Space Research Association, 3600 Bay Area Blvd., Houston, TX 77058, USA.

## Abstract

Observing the aftermath of recent impact events on the Moon tests models of impact frequency, surface change rates, and cratering physics. The Lunar Reconnaissance Orbiter Camera (LROC) Wide Angle Camera (WAC; 100-meter scale) images the whole Moon each month and has done so since 2010. Comparing images from two months under similar lighting conditions enables automated detection of surface changes. In fall 2025, a routine temporal comparison of WAC image sets revealed a new 222-meter-diameter crater that formed in spring 2024. This is the largest contemporary impact crater found in the Solar System and, according to current models, a once-in-132-year event. Later, a series of LROC Narrow Angle Camera (NAC) images provided high-resolution (meter-scale) photometric and topographic information. These images show that the crater exposed complex geology, inform current models of cratering mechanics, and document widespread surface changes extending more than 1000 crater radii.

## INTRODUCTION

The Moon serves as an exceptional natural witness plate for contemporary inner Solar System impact processes, preserving the signatures of ongoing modification in the absence of atmosphere, liquid water, and active large-scale tectonics. By leveraging temporal (before-and-after) observations, it is possible to directly detect, quantify, and interpret surface changes as they occur, providing rare insight into active regolith evolution (*1*–*3*). These datasets enable the transition from static interpretations of lunar geology to dynamic measurements of present-day processes, including impact cratering, ejecta distribution, and regolith overturn.

**Fig. 1.**
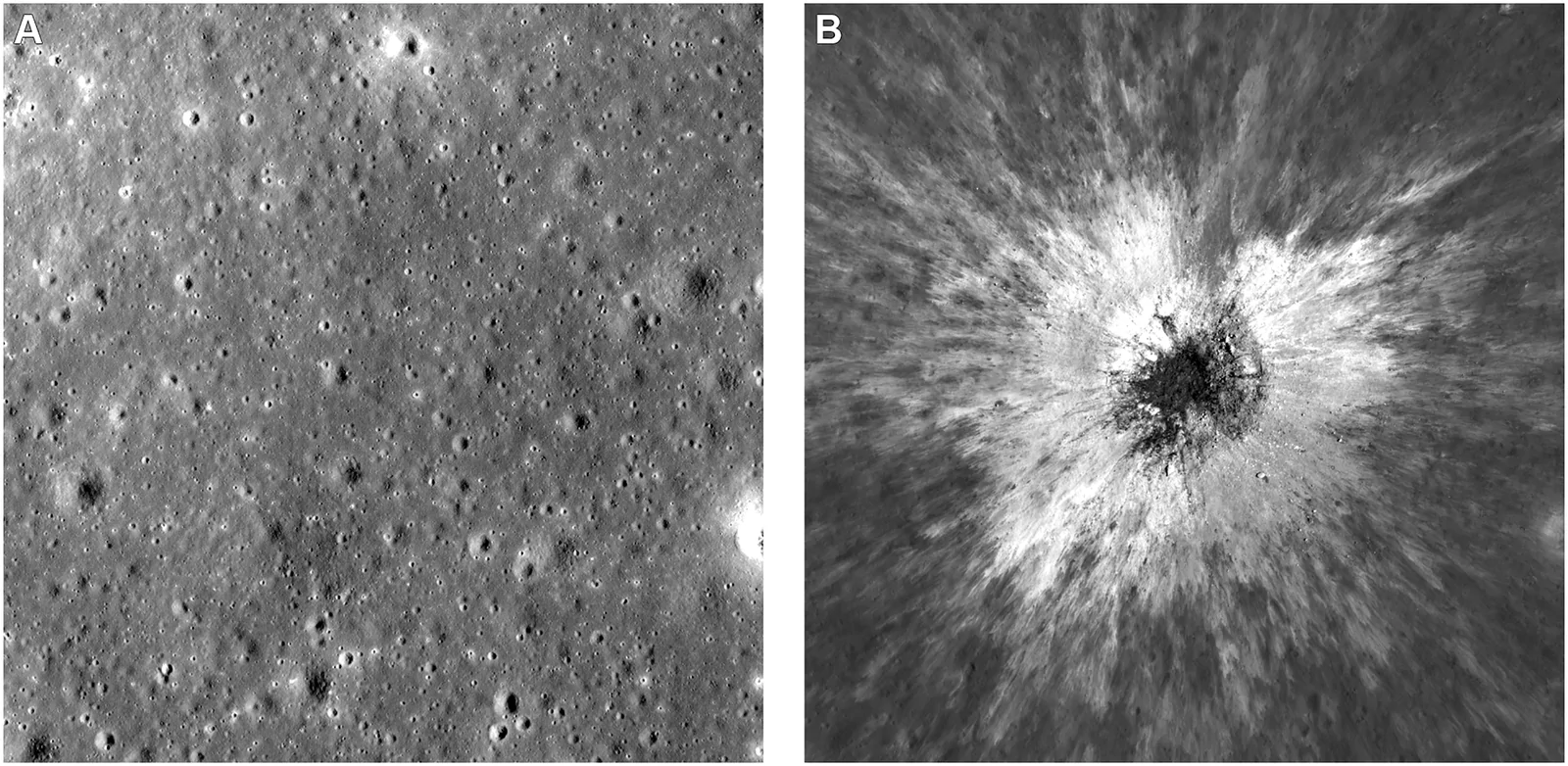
New impact site, before and after. (**A**) NAC image acquired before the impact event (M1273794308L,R). (**B**) NAC image acquired after the impact (M1521345601L). Images sampled at 1-m pixel scale; each panel is 925 m by 925 m (1.3536°N, 67.1765°E). Image acquisition parameters are listed in Table 1.

In late spring of 2024, a small cometary or asteroidal fragment impacted the Moon (Fig. 1 and Table 1), creating a 222-m diameter crater (McGetchin crater), the largest contemporary impact crater discovered anywhere in the Solar System. Prior to this event, the largest contemporary impact crater discovered on the Moon had a 70-m diameter (*3*). The magnitude of the McGetchin crater formation event (6.5 × 10^10^ kJ) represents more than an order of magnitude greater energy than that of the 70-m diameter crater event [2.4 × 10^9^ kJ; (*3*)] and more than four orders of magnitude larger than a 10-m diameter event [energy estimates assumed impactor density of 3 g/cm^3^ and an impact velocity of 15 km/s from (*4*)].

**Table 1.** Key parameters for images presented in this study. Values reported from NAC pair (L or R) that contains the crater center coordinates. Incidence, emission, phase, and subsolar azimuth angles reported in degrees.

Image	Incidence	Emission	Phase	Subsolar az.	Pixel scale (m)
M1273794308R	49	1	48	183	0.8
M146540098L	71	0	72	178	1.0
M1519004052R	8	3	6	153	0.7
M1519011064R	7	19	25	148	0.8
M1521345601L	38	2	40	169	0.9
M1527218499R	68	55	15	187	1.5
M1523687730L	69	8	77	170	0.9

Crater production models indicate that an impact of this magnitude is expected to occur only once every 132 years on the Moon (*5*). As such, this event constitutes a statistically rare, effectively once-in-a-lifetime observation. High-resolution, multitemporal datasets (images acquired before and after the impact) provide the opportunity to quantify the spatial extent, magnitude, and temporal evolution of surface modification associated with a recently formed large, fresh lunar crater. In particular, this event enables new constraints on ejecta emplacement, boulder distribution, rim uplift, and distal surface alteration.

## RESULTS

### Geologic setting

McGetchin crater formed on or near a highland-mare contact [NpNt/Im2 in (*6*) and Nt/Im2 in (*7*); 1.3536°N, 67.1765°E, −846 m elevation before the impact] just inside the outer rim of the Crisium basin [1080 km ring identified by (*8*)]. The site lies 330 km from the edge of Mare Crisium where mare materials embayed the southeastern flank of Dubyago N crater (7200-m diameter). The embaying mare is characterized by relatively thin flows that flooded hummocky highland terrain, leaving an irregular contact with numerous highland kipukas. The surface of Mare Spumans (1.32°N, 66.52°E, −1830 m elevation), 20 km west of the new crater, is about 1000 m lower than the new crater. The impact location provides an opportunity to investigate how complex target properties (mare-highland contact) affect crater formation and ejecta emplacement.

**Fig. 2.**
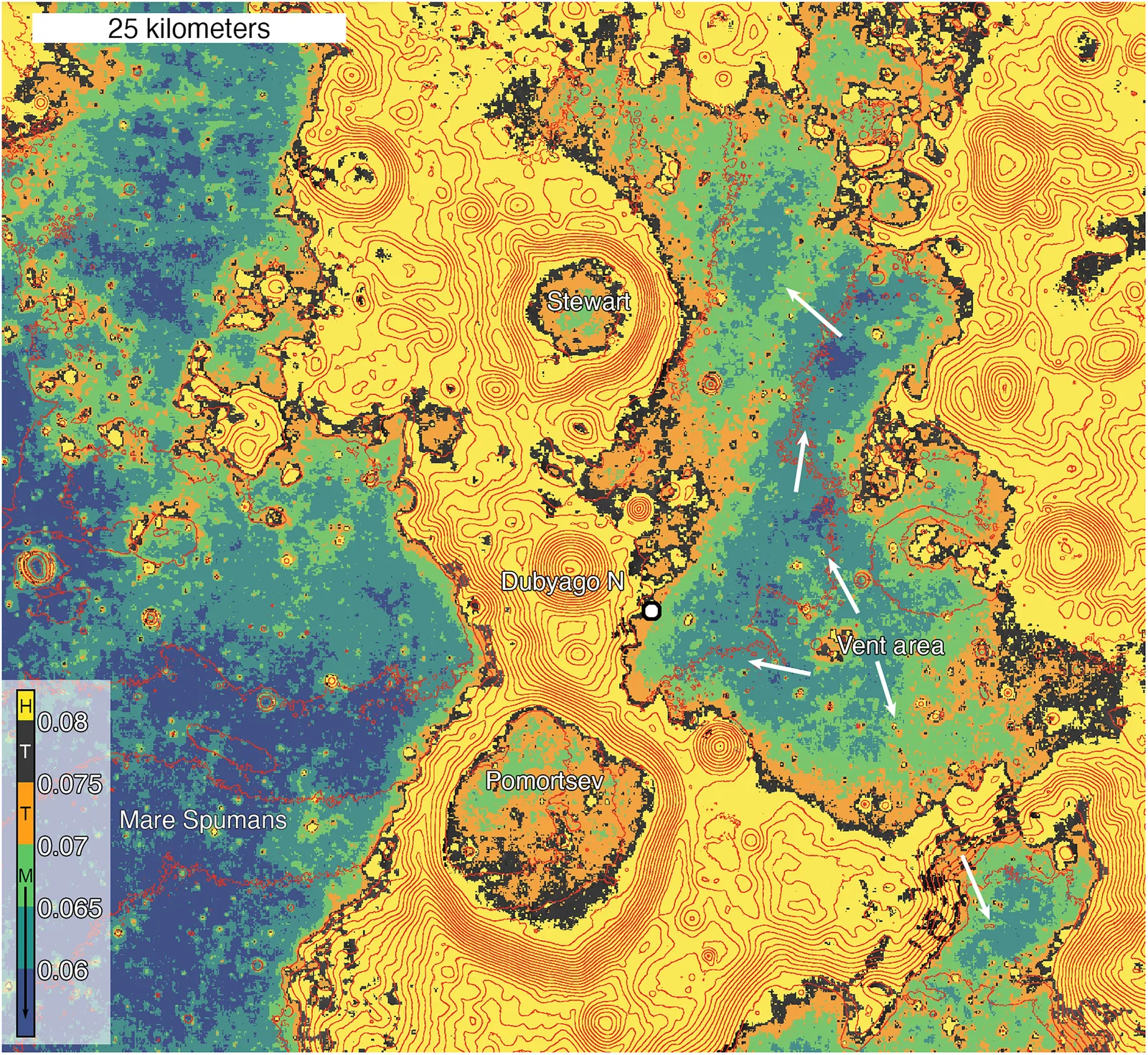
Regional geologic context map. Basemap colors represent WAC normalized reflectance [30° incidence angle, 0° emission angle, and 30° phase angle; (*30*)], 100 m topographic contours (red) from SLDEM (*31*). Shades of blue and green are mare materials (M), orange and black (T) indicate transition from mare to highland regolith materials, and highland (H) terrain is yellow. Arrows indicate the direction of the mare basalt flows from the vent area. White dot southeast of Dubyago N crater indicates McGetchin crater location. The map is 184 km wide, north is up.

A local high point (1.246°N, 67.935°E, −730 m elevation) within the adjacent mare materials is interpreted as an ancient eruptive center and occurs 23 km ESE of the new crater. From the eruptive center, basalt flowed to the northwest, met a topographic high, and was diverted to the northeast, and then again to the northwest (Fig. 2). Basalt also flowed to the south from the same eruptive center, descending a steep slope (>10°) in two places and continuing down a shallow slope (0.600°N, 68.197°E, −1520 m elevation), to just north of McLaurin O crater (0.009°N, 67.788°E, −1688 m elevation), 45 km SSE of McGetchin crater. The complex topographically controlled small-scale mare eruptions are similar in size and elevation to the high-standing mare puddles around the Orientale basin: Lacus Autumni (−300 m) and Lacus Veris (−700 m) (*9*).

### Crater morphology

**Fig. 3.**
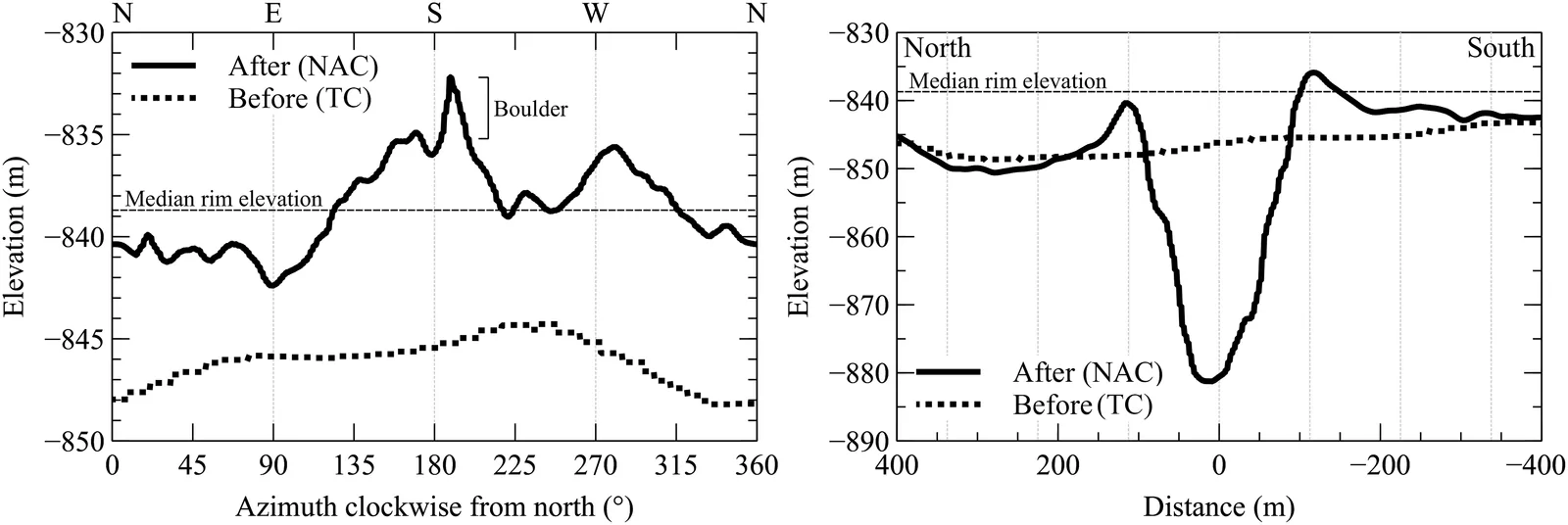
Before and after impact topographic profiles. Left: Topographic profile tracing elevation values around the crater rim (see dashed circle indicating rim trace in Fig. 5) and the corresponding pre-impact topography. The sharp peak at 190° azimuth is a large boulder perched on the crater rim. Right: North-to-south elevation profile through the crater center. Vertical lines mark intervals of one crater radius (111 m). Differences between the before and after profiles beyond one crater radius from the rim are likely due to uncertainty in the Kaguya TC DTM.

We measured basic crater parameters from a Lunar Reconnaissance Orbiter Camera (LROC) (*10*) Narrow Angle Camera (NAC) stereo-based Digital Terrain Model (DTM) (*11*) at 3 m/pixel (better than 1.5-m vertical precision) made from images taken after the impact, corresponding 1 m/pixel orthomosaics, and a preimpact Kaguya Terrain Camera (TC) (*12*) DTM at 22 m/pixel (3-m vertical precision) (*13**,* *14*). The crater diameter ranges from 213 m in an ESE (101°) direction to 231 m in an NNE (011°) direction (222-m average diameter). We defined the rim as the point where the directional slope is closest to zero moving away from the crater geometric center (1.3536°N, 67.1765°E, measured on NAC_DTM_NEW200CRAT3), switching from positive inside the crater to negative on the ejecta. From this rim traverse, we extracted elevation values and found a minimum of −842 m, a maximum of −832 m (−835 m after excluding a large boulder that straddles the southern rim; Fig. 3), and a median of −839 m. The lowest point within the crater (−882 m, 1.3540°N, 67.1763°E) occurs 15 m NNW (334°) of the crater geometric center, yielding a depth of 43 m (median rim elevation minus the maximum depth) and a depth to diameter (*d*/*D*) ratio of 0.19 (Fig. 3 and Table 2), typical of the freshest craters of this size (*15*).

**Table 2.** Key geomorphic parameters of the new crater. The rim height was derived by subtracting a median value of the preexisting terrain from the Kaguya DTM from the NAC derived DTM (Fig. 3). SD, standard deviation.

	Value	Model	Model uncertainty	Model Reference
Diameter minimum (m)	213			
Diameter maximum (m)	231			
Average diameter, *D* (m)	222			
Interior slope (°; median, SD)	24, 9.5			
Maximum interior slope (°)	41			
Rim height (m; median, SD)	7.7, 2.0	7.8		(*16*)
Rim uplift (m)		3.3		(*17*)
Ejecta thickness at rim (m)		5.3	±1.7	(*32*)
Ejecta thickness at rim (m)		4.6		(*17*)
Largest ejected boulder (m)	11	8	±4	(*33*)
Depth, *d* (m)	43	43		(*15*)
*d*/*D*	0.19	0.19		(*15*)
Limit of continuous ejecta from crater center (m; median, minimum, maximum)	259, 126, 360	268	−52, +64	(*33*)

**Fig. 4.**
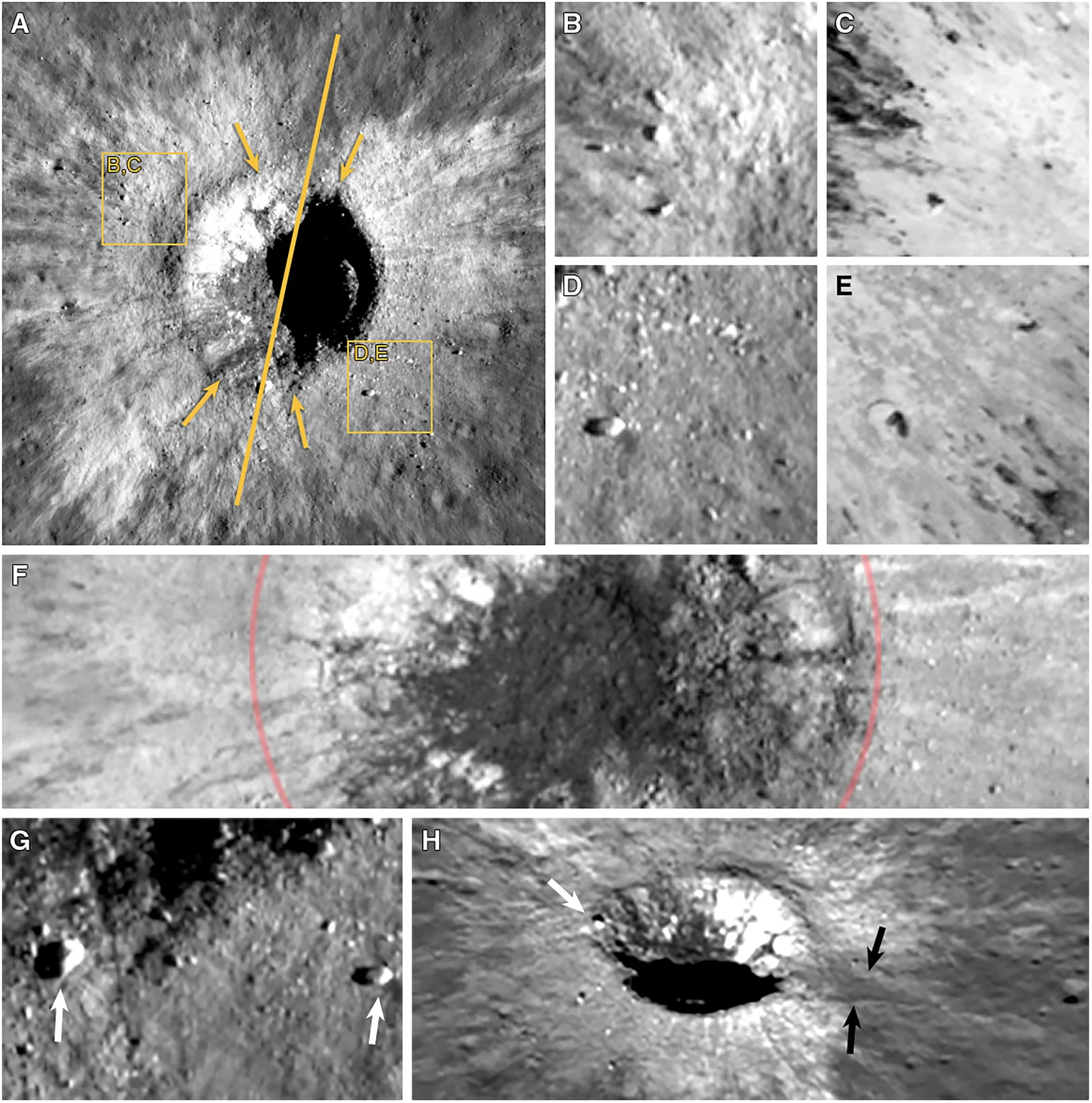
Key geomorphic features of McGetchin crater. (**A**) Arrows indicate topographic discontinuities delimiting western and eastern arcs. The line traverses a 12° orientation from north, aligned with vectors between the ends of the arcs. The upper-left box indicates the location of (B) and (C); the lower-right box indicates the location of (D) and (E). Image is 578-m wide, north is up; NAC M1523687730L,R. (**B** and **C**) Same areas on the NW ejecta with incidence angles 69° (B, M1523687730R) and 7° (C, M1519004052R). (**D** and **E**) Same areas on the SSE ejecta with the same incidence angles, all four panels enlarged three times, 90-m wide. (**F**) Central portion of the crater and proximal ejecta (enlarged three times), incidence angle 38°, 385-m wide; red arcs indicate crater rim (M1521345601L). (**G**) Enlargement (three times) of southern rim; left arrow indicates largest boulder (13 × 9 × 3 m), and right arrow indicates second largest boulder (8 × 7 × 2 m), also shown in (D) and (E) (panel is 142-m wide, M1523687730L). (**H**) Oblique view of crater viewed from east to west. The white arrow indicates the largest boulder [see also (G)]; the black arrows mark the northern tongue. The image is about 750 m wide, and north is approximately to the right (M1527218499R).

**Fig. 5.**
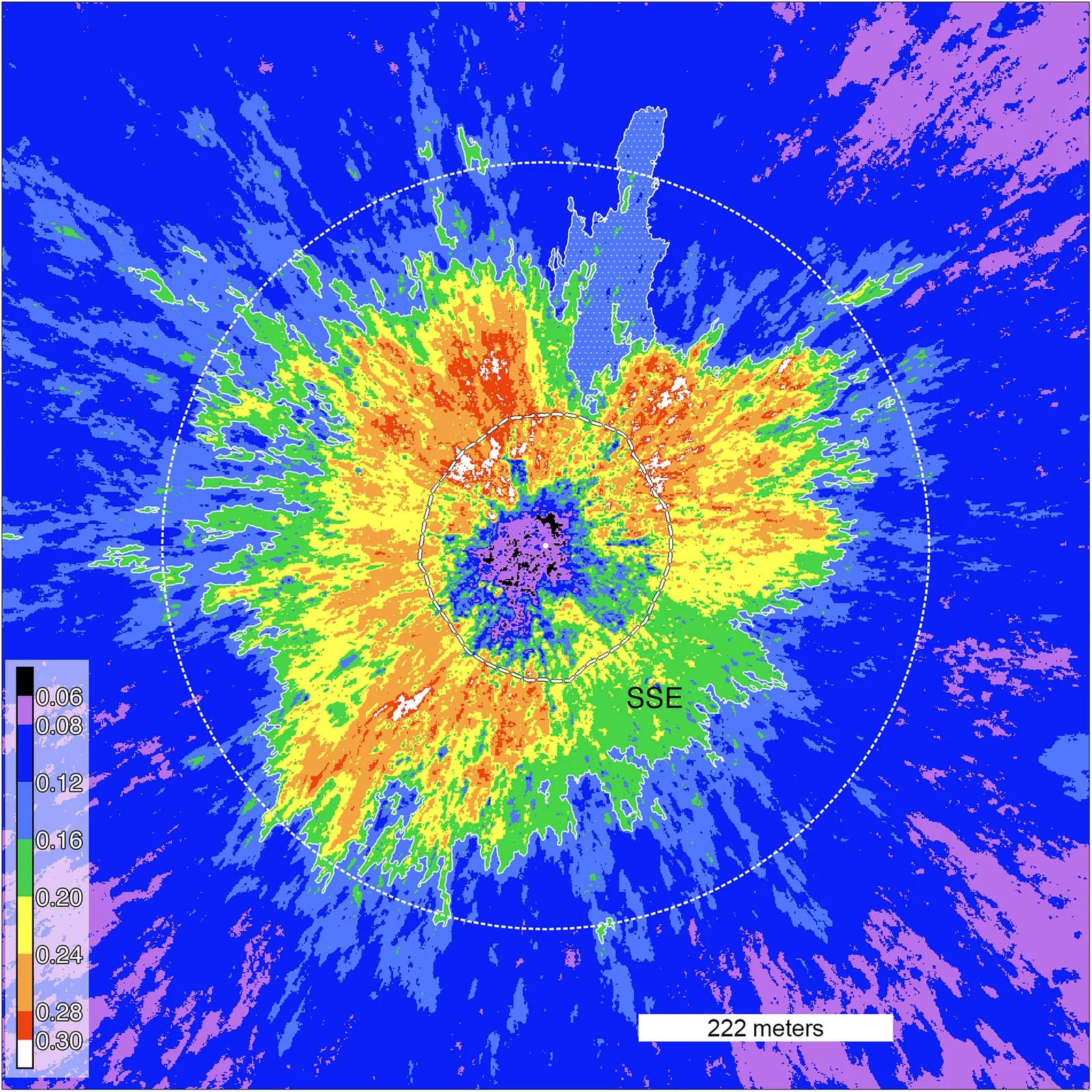
Color-coded binned reflectance values. Inner circle (dashed line) traverses crater rim, and outer circle indicates 2-crater radii from the crater rim. Outer edge of the >0.16 unit highlighted with white outline (interpreted as edge of continuous ejecta), northern tongue unit indicated with stipple pattern, and SSE locates the intermediate reflectance ejecta unit. Image width is 950 m, north is up, NAC M1519011064L,R (7° incidence, 19° emission angle, 26° phase angle).

We analyzed the TC DTM relative to the NAC DTM to estimate the rim height. After aligning the TC DTM to the NAC DTM, we subtracted the TC DTM from the NAC DTM rim traverse, yielding a median rim height of 7.7 m and a standard deviation of 2.0 m (*n* = 709 points), which is consistent with the 7.8-m rim height predicted by the relation presented in (*16*). The maximum relief along the rim is 13 m, but this occurs across the boulder perched on the rim (Figs. 3, 4, and 5); subtracting the boulder height brings the maximum rim height to 10 m (Table 2). We estimated ejecta thickness at the rim (4.6 m) using a relation derived from terrestrial crater measurements and estimates from lunar craters (*17*). From this value, the estimated structural uplift at the rim (subtracting the ejecta thickness estimate from the median rim height measurement) is 3.1 m or 40% of the rim height measurement (Table 2).

The rim is bisected in an NNE direction, delimited by rim discontinuities on both the north and south sides of the crater (Fig. 4). The western arc of the rim has a larger diameter than the eastern arc (230 versus 214 m, extrapolated from each arc to a full circle). The geometric centers (1.3535°N, 67.1767°E and 1.3535°N, 67.1766°E) of these two fit circles are within 5 m (4.3 m from each other and 6.6 and 2.4 m from the crater center, respectively). Since the crater is not perfectly circular, we estimated the minimum and maximum diameters by fitting an ellipse to the rim traverse points. The maximum diameter (231 m) occurs at 011° azimuth, and the minimum (213 m) at 101° azimuth. We adopt the midpoint between the minimum and maximum diameters as the average diameter (222 m).

The crater interior is funnel-shaped, with a small (15 × 30 m) hummocky floor and a 16 × 32 m slump boulder that occurs roughly 10 m below the eastern rim. The crater walls are steep with a median wall slope of 24° and a maximum slope of 41° (computed on a 12-m baseline).

### Crater interior reflectance

The crater interior exhibits a reflectance contrast >7 (*I*/*F* 0.045 to 0.34; incidence 7°, phase 25°, NAC ID M1519011064L,R; Fig. 5). The rough, hummocky depths of the crater exhibit distinctly low reflectance (<0.090); a streak of this low-reflectance material also occurs as splotches on the southern wall. The lowest reflectance material (0.045) is lower than distant mature mare material (>0.050). We interpret this material as enriched in glassy, quenched impact melt, perhaps of mare origin. There is no sign of pooled impact melt within the crater, consistent with the “impact veneer” class of (*18*) (see figure 8 therein). The highest reflectance value (0.34) exceeds those of all other areas in the 9 × 27 km NAC image; only three small splotches (perhaps eroded boulders) within a degraded crater at 1.047°N, 67.138°E have reflectance >0.30.

### Proximal ejecta reflectance

The low reflectance material found in the crater interior is absent in the ejecta blanket. The higher reflectance ejecta (>0.16) lies primarily within two crater radii of the crater rim, with an outer edge ranging from 126 to 360 m from the crater center (Fig. 5), and the median extent is 258 m (1.32 radii from the crater rim). The highest reflectance materials (>0.30) occur on either side of a narrow tongue of intermediate reflectance material (0.12) that emanates from the northern rim (014°), and in a small patch on the SSW ejecta blanket (white unit, Fig. 5). We note alignment between the northern tongue, the SSW higher-reflectance material, and the largest diameter of the crater, which may indicate the angle and direction of impact or perhaps a discontinuity in target material properties. The tongue appears to be composed of granular material rather than impact melt, most likely the result of ejected basaltic material; however, its origin remains enigmatic. A distinctive intermediate reflectance unit (0.16 to 0.20) occurs just beyond the rim between 113° and 178° azimuth (Fig. 5, SSE) and may indicate excavated mare material admixed with highland regolith. We searched for, but did not find, definitive evidence that the bolide impacted at a grazing angle (e.g., an elliptical crater shape or a forbidden zone in the ejecta).

### Distal disturbance zones

The LROC Wide Angle Camera (WAC) acquired an average of 540 observations for each pixel location over the target region prior to the impact event, and an additional 60 observations following the event. By photometrically normalizing all pre- and post-impact images to a common photometric geometry and co-registering them into composite mosaics, we significantly enhanced the signal-to-noise ratio relative to individual observations. This stacking approach suppresses stochastic noise while preserving coherent surface reflectance signals, enabling the detection of subtle, spatially extensive reflectance variations (*3**,* *19*).

**Fig. 6.**
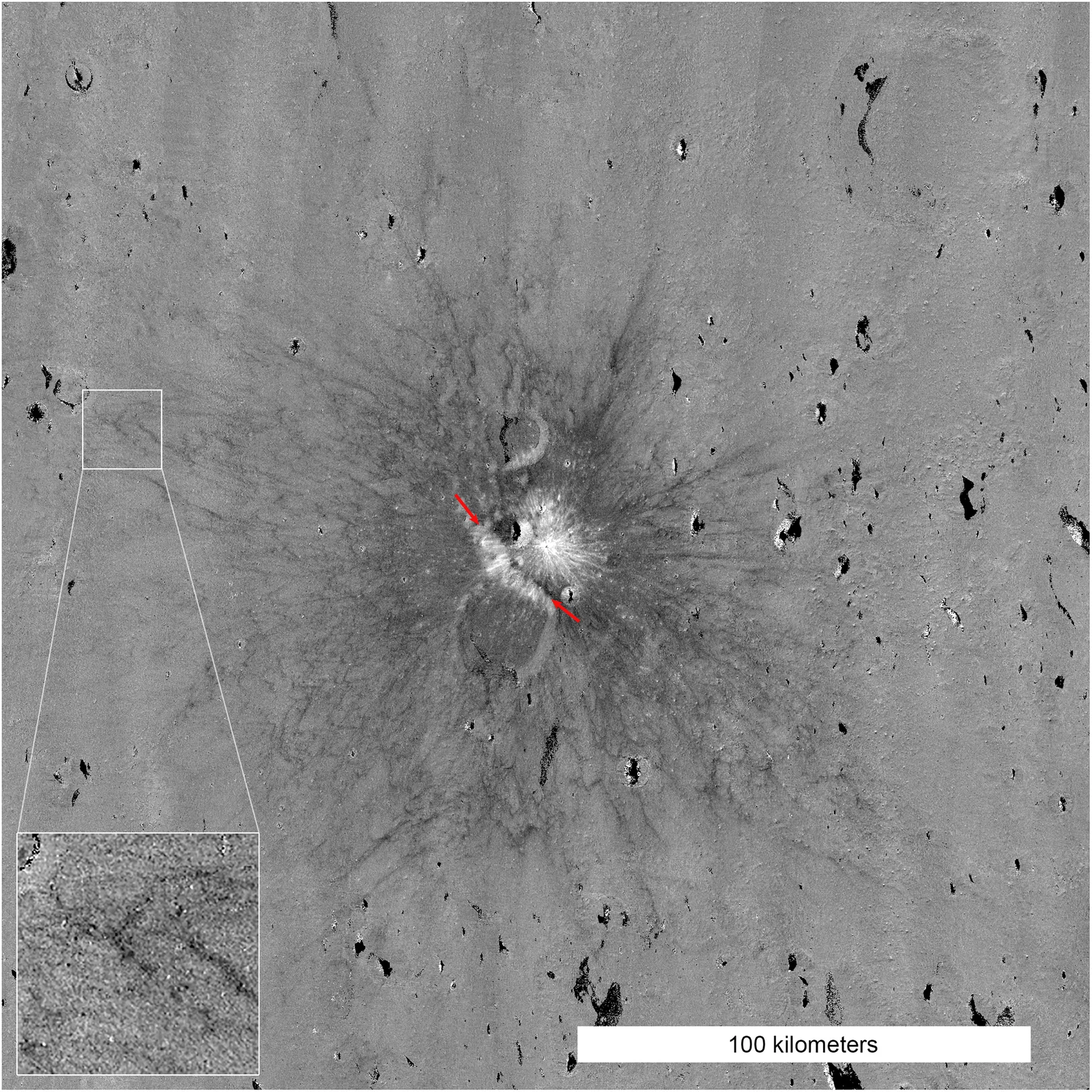
WAC temporal ratio map (after/before). Temporal ratio map from mosaic constructed from an average (per pixel) of 540 LROC WAC observations acquired prior to the impact and 60 observations acquired afterward. The ratio image highlights the extent of the ejecta disturbance, including a distal high-reflectance zone extending ∼15 km from the impact site (image center) and a broader distal low-reflectance zone reaching 100 km, with localized disturbances extending to ∼140 km (detail enlarged three times). Ridgeline west of the new crater formed by topography related to Dubyago N and Pomortsev craters (red arrows) would have served as an obstacle to low-angle ejecta; note the apparent landslides on the steep western side of the ridge. Black areas represent steep slopes with significant shadowing (nulled); adjacent bright areas are also topographic artifacts. Image width is 240 km; north is up; 689 nm band; corrected to incidence 30°, emission 0°, and phase 30°; ratio values stretched from 0.94 to 1.06 to 0 to 255 in the main image. Enlargement panel further contrast-stretched 48 to 210, and 0 to 255.

Ratioing the post-impact mosaic to the pre-impact mosaic isolates impact-induced surface changes, revealing faint reflectance anomalies (∼1%) that extend well beyond the immediate ejecta blanket. In particular, we identify a distal high-reflectance zone (DHRZ) that extends to radial distances of 15 km from the impact site and a broader discontinuous low-reflectance zone (DLRZ) that reaches >120 km beyond the DHRZ (Fig. 6). The distal disturbances can be found more than 1000 crater radii from the new crater and in some cases exhibit a delicate lacey texture (see enlargement panel in Fig. 6). The lace-like texture probably formed as clumps of ejected material separated and spread out over the longer flight times required to reach distal areas. Upon impact, these sparse particles churned the epiregolith, magnifying their imprint. Closer to the crater, the ejected material was denser, and impact churning effects overlapped.

Distal reflectance zones have been documented at other young impact sites (*1*, *2*). For example, an ∼70-m-diameter crater that formed around October 2012 and was analyzed in (*3*) exhibits a DLRZ extending to ∼100 km from the crater, suggesting that these disturbance zones are a common outcome of small-to-moderate lunar impact events.

The two distal zones may have been generated by impact-produced vapor phases (that accelerated fine particles of granular materials and melt) released early in the cratering process (*2**,* *3**,* *20**,* *21*). As the vapor plume expanded radially across the surface (at a low angle), it interacted with the uppermost regolith, mobilizing fine particles and inducing microscale textural modifications. This process likely modified the effective roughness of the optically active epiregolith, altering its photometric response. Speyerer *et al.* (*2*) proposed that the DHRZ was smoothed by jetting, a high-velocity, low-angle initial ejection of material, thereby reducing roughness and increasing reflectance. Likewise, they proposed that the DLRZ was roughened by the jetting process, resulting in increased shadowing and lower reflectance. In the latter case, an increased volume of melt and clastic material churned the top few millimeters, increasing roughness.

Due to the complex topography around the impact site, the new crater provides an opportunity to inform models of jetting and surface disturbance. Eight kilometers west of the crater lies a ridge (700 m above the crater) that drops off steeply (>12°) to the west down to Mare Spumans (1000 m below the crater). Here (14 km from the crater), the mare reflectance is up to 2.5% lower than before the impact, similar to other areas of the DLRZ at the same distance, indicating equivalent surface disturbance despite the potentially shielding ridge; ballistic particles would have had to be ejected at angles greater than 13° to clear the ridge and impact at its base. To reach the more distant discontinuous DLRZ, the ejection angle would have to be >7° to clear the ridge.

The conventional wisdom (*21*) is that jetting results in particles ejected at very low angles relative to the surface (<2°) at very high velocities (>0.5 times the bolide impact velocity). If the DLRZ at the base of the ridge was formed from jetted particles, perhaps an elongate (versus a spherical) bolide impacted, permitting a wider range of ejection angles and velocities. Alternatively, perhaps escaping gas-particle collisions can efficiently disperse the jetting trajectories, allowing particles to reach the base of the ridge. Alternatively, two ejection regimes may have produced the more distal discontinuous DLRZ and the continuous portion (closer to the ridge) of the DLRZ. We interpret that the distal patterns formed as a result of the contact and compression (jetting) stage and not the excavation stage from which rays are derived. Detailed modeling of the formation of the distal ejecta patterns (from 10 to >100 km distance from the crater; Fig. 6) could advance our understanding of the now poorly understood details of the contact and compression phase of impact events (*21*).

### Ejected boulders

**Fig. 7.**
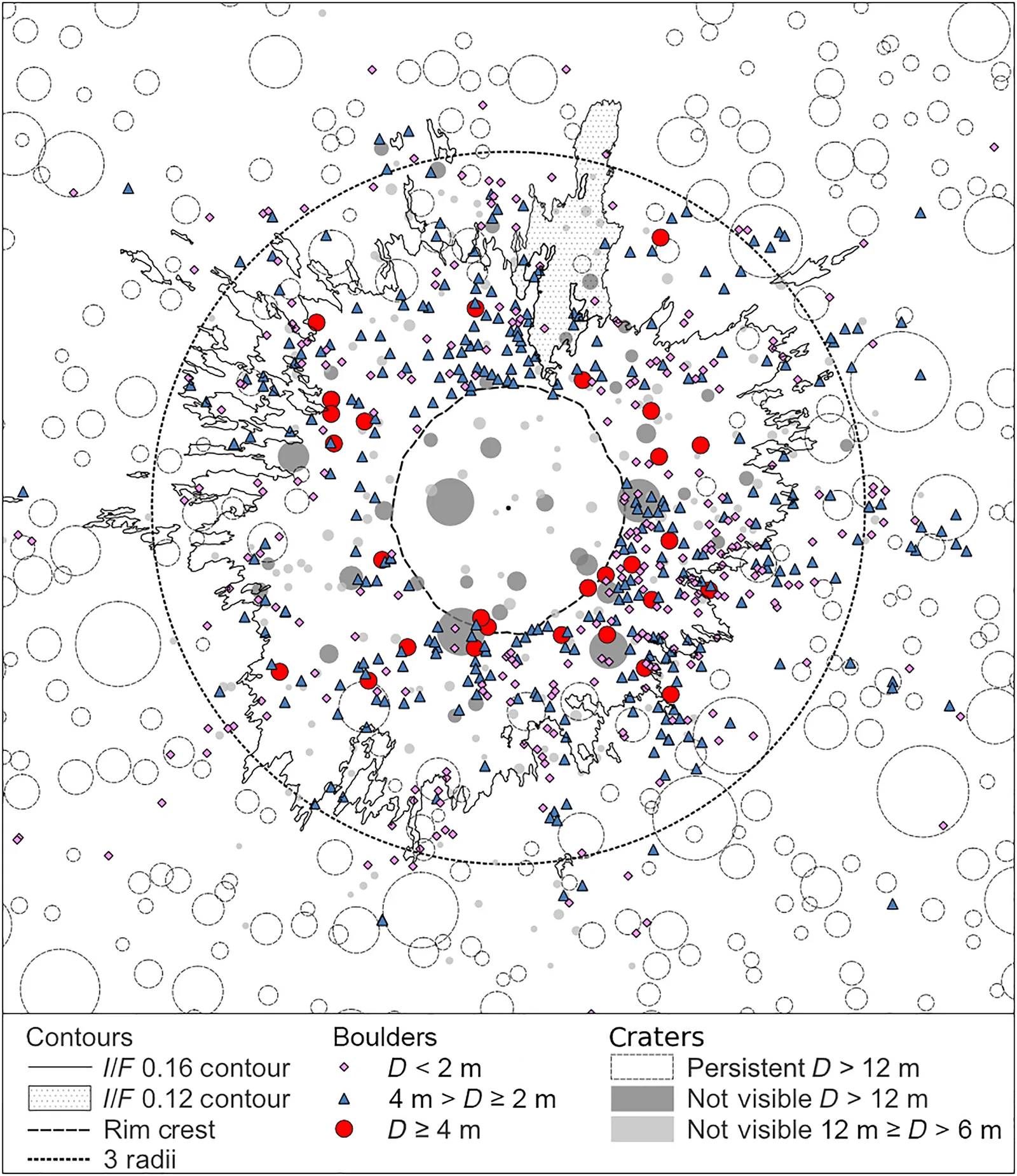
Geomorphic map centered on McGetchin crater. Solid black line indicates interpretive boundary of continuous ejecta (see Fig. 5), crater rim delimited with long-dash black line, 2-crater radii from rim shown with short-dash black line. The lower-reflectance northern tongue is indicated by a stippled pattern. Craters that existed before the impact event and are still identifiable are indicated with dotted circles (only diameters >12 m shown); craters no longer identifiable post-impact are indicated with filled circles (light gray diameters between 6 and 12 m diameter, dark gray craters greater than 12 m diameter). Boulders (all formed as a result of the McGetchin impact event) are color-coded by diameter (boulders digitized from a 1-m sampled image, M1523687730); map width is 950 m; north is up.

Boulder concentrations (Fig. 7 and figs. S1 and S2) occur on opposite sides of the northern tongue (344° and 040° azimuth) (Fig. 4). The largest concentrations of ejected boulders occur in two arcs on the south flank, between 090° and 145° (contained within the SSE intermediate reflectance unit) and 180° to 210° azimuth. The two largest boulders are found on the southern rim (190° azimuth) and on the SE flank (130° azimuth) and measure 13 × 9 × 3 m and 8 × 7 × 2 m (Table 2). We estimated boulder heights (3.2 ± 0.4 m and 1.5 ± 0.4 m, respectively) from shadow measurements on a NAC image acquired with an incidence angle of 69° (M1523687730L). Maximum ejected boulder sizes for terrestrial and lunar craters were shown to follow a power-law relation (*22*); that relation predicts a maximum boulder size of 8 m (4 to 12 m range), consistent with the average diameter of the largest boulder here (11 m). The majority of boulders (86%, *n* = 25) with diameters >4 m are found within the 0.16 reflectance boundary (Figs. 5 and 7). These results are consistent with studies of boulders around lunar craters presented in (*23*) and (*24*).

Small low reflectance zones (−30%; M1519011064, Fig. 4E) occur down slope from several larger boulders (Fig. 4, B to E). The origin of these reflectance zones is not clear. Several hypotheses may explain their formation. During emplacement, a boulder may form a barrier for trailing granular material. Alternatively, a ballistically ejected boulder may have excavated lower reflectance material upon impact. A third possibility is that late-stage, low-angle, high-reflectance material may be “shadowed” and prevented from landing downhill of a boulder. Each of these possibilities carries different implications for understanding ejecta emplacement: The granular flow barrier hypothesis suggests significant ground flow and mixing of ejecta; the ballistic excavation hypothesis indicates that boulders are very late-stage components of ejection and their ejecta only spreads out along the boulder momentum vector; and the shadow hypothesis necessitates a low-angle high-reflectance component that trails the majority of ejecta emplacement.

### Erasure of preexisting craters

The preexisting target was Imbrian-aged mare materials, thus heavily cratered (Fig. 7 and fig. S3). The largest preexisting crater within the bounds of the new crater was 40 m in diameter, and the largest between the new crater rim and the two-radii mark was 60 m in diameter. There are no signs of preexisting craters within the new crater. One of the largest preexisting craters was situated along the SSW rim (∼190° azimuth), and no traces of its existence are found; this location is where the rim reaches its greatest height. Outside the rim and within the 0.16 reflectance boundary, only five of the 103 preexisting craters with diameters ranging from 6 to 12 m survived, while 12 of the 35 craters with diameters ranging from 12 to 48 m survived the impact. All surviving craters are heavily degraded, and the largest are found within an arc stretching from 170° to 280°.

## DISCUSSION

We interpret the continuous ejecta margin as defined by the outer edge of reflectance (*I*/*F*) values exceeding 0.16 (Figs. 5 and 7). Most preexisting craters within this boundary are no longer identifiable, whereas those outside the boundary are predominantly readily identifiable. The median distance from the rim to the edge of the 0.16 reflectance unit is 146 m (1.3 radii); from the equation of (*22*) (for craters larger than 650-m diameter), a 222-m crater should have a continuous ejecta deposit extending 157 m (1.4 radii; −52 m, +64 m uncertainty) from the rim. Due to the enigmatic nature of the northern tongue, we excluded it from our calculation of the distance to the edge of the continuous ejecta. Additionally, the majority of boulders >4 m in diameter are contained within the reflectance boundary. In reality, the boundary is diffuse and does not follow a strict reflectance cutoff; however, the above observations are consistent with a delimited continuous ejecta zone more-or-less coincident with the edge of the 0.16 reflectance cutoff.

**Fig. 8.**
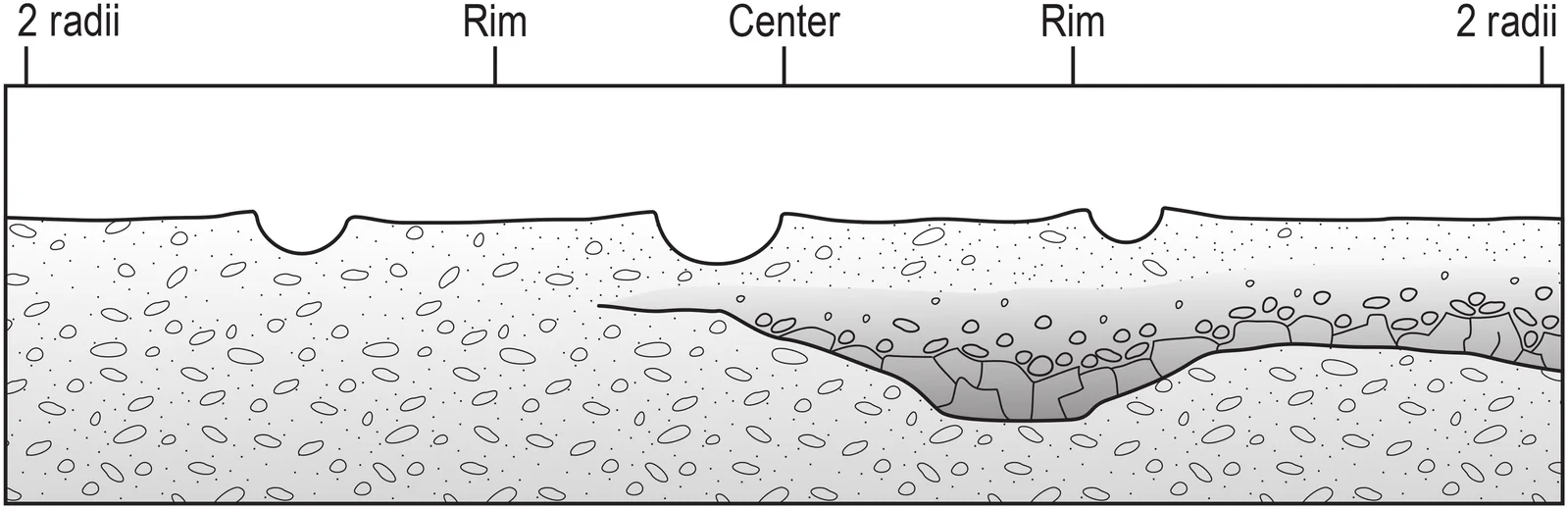
Interpretive cross-section of pre-impact stratigraphy. We interpret the crater to have formed where mare flows (darkly shaded) lapped up against the highland flank of Dubayago N crater. A highland regolith lies under and around the mare materials. The mare unit is of Imbrian age (*6*), so a regolith of ∼10 m thickness occurs above the mare, and due to vertical and horizontal mixing, it comprised mixed highland and mare components. West is to the left and east is to the right.

The general crater morphology follows expected crater formation scaling patterns: *d*/*D*, rim height, largest ejected boulder, and continuous ejecta extent (Table 2). However, the crater is not completely law-abiding. The rim deviates from circular, with rim discontinuities aligned 12° east of north. This alignment is shared by several landforms: a boulder cluster on the south rim, the northern lower reflectance “tongue,” the eastern slump boulder, and the south-southeast ejecta lower reflectance zone with its colocated boulder field although there are no traditional indicators of an oblique impact along this azimuth (e.g., elliptical crater and forbidden zone in ejecta). These occurrences are interpreted as resulting from the impact straddling a mare-highland contact. More specifically, the greater diameter on the western arc suggests excavation within a loose granular regolith, while the smaller diameter of the eastern arc suggests a coherent subsurface lava flow (*25*). Over time, impact-induced vertical and horizontal mixing of thin mare materials emplaced over a highland regolith (Fig. 8) could have produced the intermediate reflectance values (Fig. 5, SSE) excavated and emplaced here; the mare component would increase with depth and likely occupies a significant portion of the 20-m depth of excavation. Additionally, the highest boulder density occurs in this ejecta unit, consistent with bouldery mare material at depth.

Surface disturbances from distal ejecta extend >120 km (1000 crater radii), which is thought to be caused by high-speed jetting (*2*, *3*) that occurs during the contact/compression phase of the impact process. While jetted material is assumed to be escaping the impact site at low-angle trajectories, we can see in this example that the jetted material must have cleared the western ridge and carried enough material to produce a continuous lower reflectance region on the mare at the western base of the ridge and the floor of Pomortsev crater. After passing these topographic obstacles, the remaining jetted material would have had to retain sufficient energy to modify surface reflectance over a 100-km distance. Current knowledge of the jetting process is incomplete (*20**,* *21*), and the distal disturbances documented here provide the means to improve jetting models.

Exploration of this crater by a mobile surface asset would greatly enhance our knowledge of cratering mechanics and the photometric response of newly disturbed materials and would provide a baseline for investigating rates of change associated with regolith evolution (*1*–*3*). In situ measurements of surface properties would also provide insight into the nature of “cold spot” thermal anomalies observed around young Copernican and contemporary craters >30 m in diameter (*26**–**29*). Together, these measurements would directly link impact processes to the physical and thermal evolution of the upper regolith, thereby improving our ability to interpret orbital observations and constrain regolith evolution models.

## MATERIALS AND METHODS

All materials and methods are explicated in the text.
