# Supplementary material for: A new 222-m diameter lunar crater
Source: Sci Adv. 2026 Sep 16;12(38):eaeh7812. doi: 10.1126/sciadv.aeh7812 (PMC13580579; doi:10.1126/sciadv.aeh7812)
Supplement: Supplementary file 1 [file sciadv.aeh7812_sm.pdf]

Science Advances
AAAS

# Supplementary Materials for

## A new 222-m diameter lunar crater

Mark S. Robinson *et al.*

Corresponding author: Mark S. Robinson, mrobinson@intuitivemachines.com

*Sci. Adv.* **12**, eaeh7812 (2026)
DOI: 10.1126/sciadv.aeh7812

**This PDF file includes:**

Figs. S1 to S4

**Supplemental Materials**

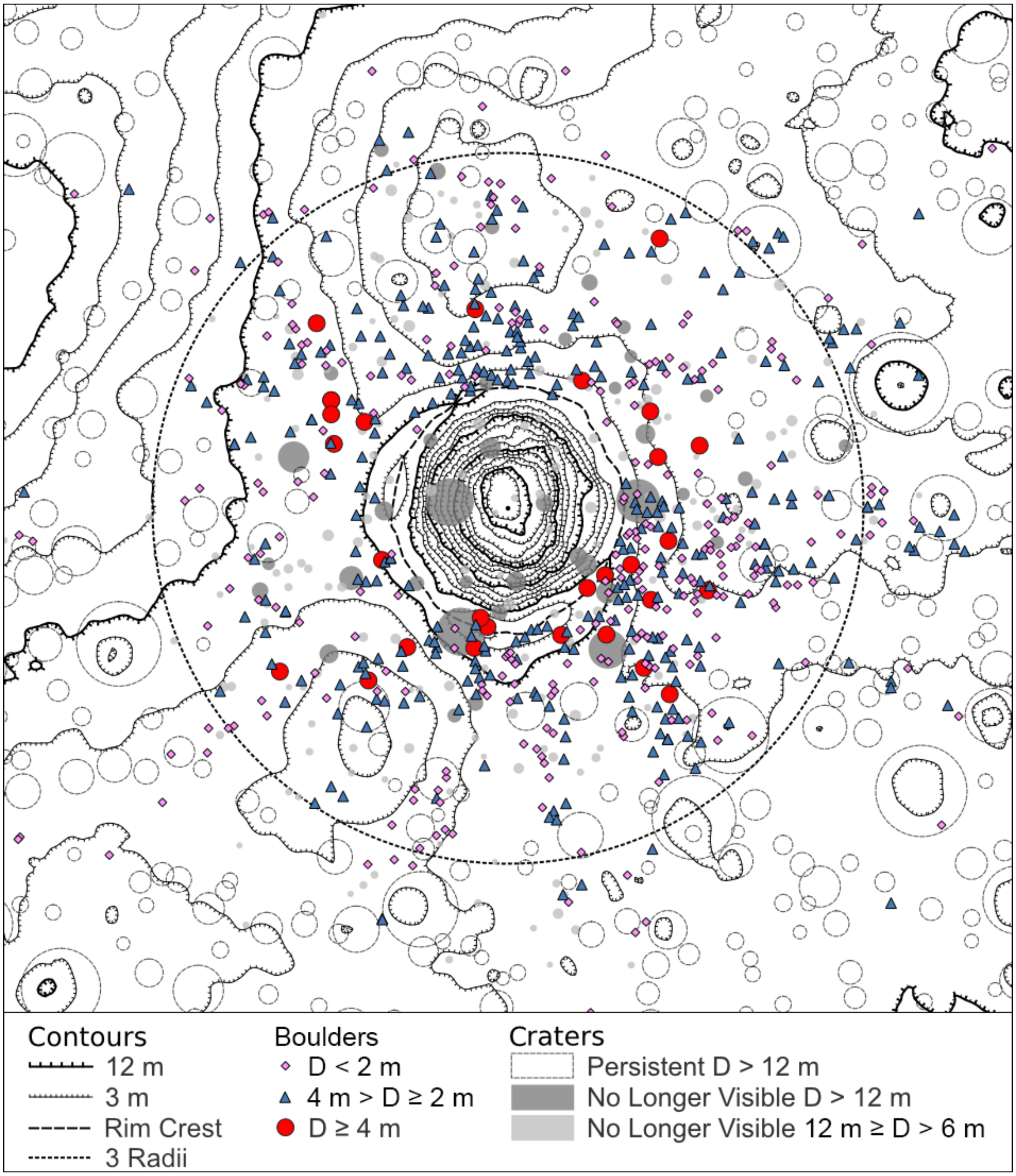


**Supplemental Figure 1. Topographic map centered on McGetchin crater.** Major contour interval 12 m, minor contour interval 3 m, tick marks on contours indicate downhill direction. The inner dashed line indicates the crater rim, the outer dashed line indicates a 3-radii range from the crater center. Craters that existed before the impact event and are still identifiable are indicated with dotted circles (only diameters >12 m shown); craters no longer identifiable post-impact are indicated with filled circles (light gray diameters between 6 m and 12 m diameter, dark gray craters greater than 12 m diameter). Boulders (all formed as a result of the McGetchin impact event) are color-coded by diameter

(boulders digitized from a 1 m sampled image, M1523687730L); map width is 950 m; north is up.

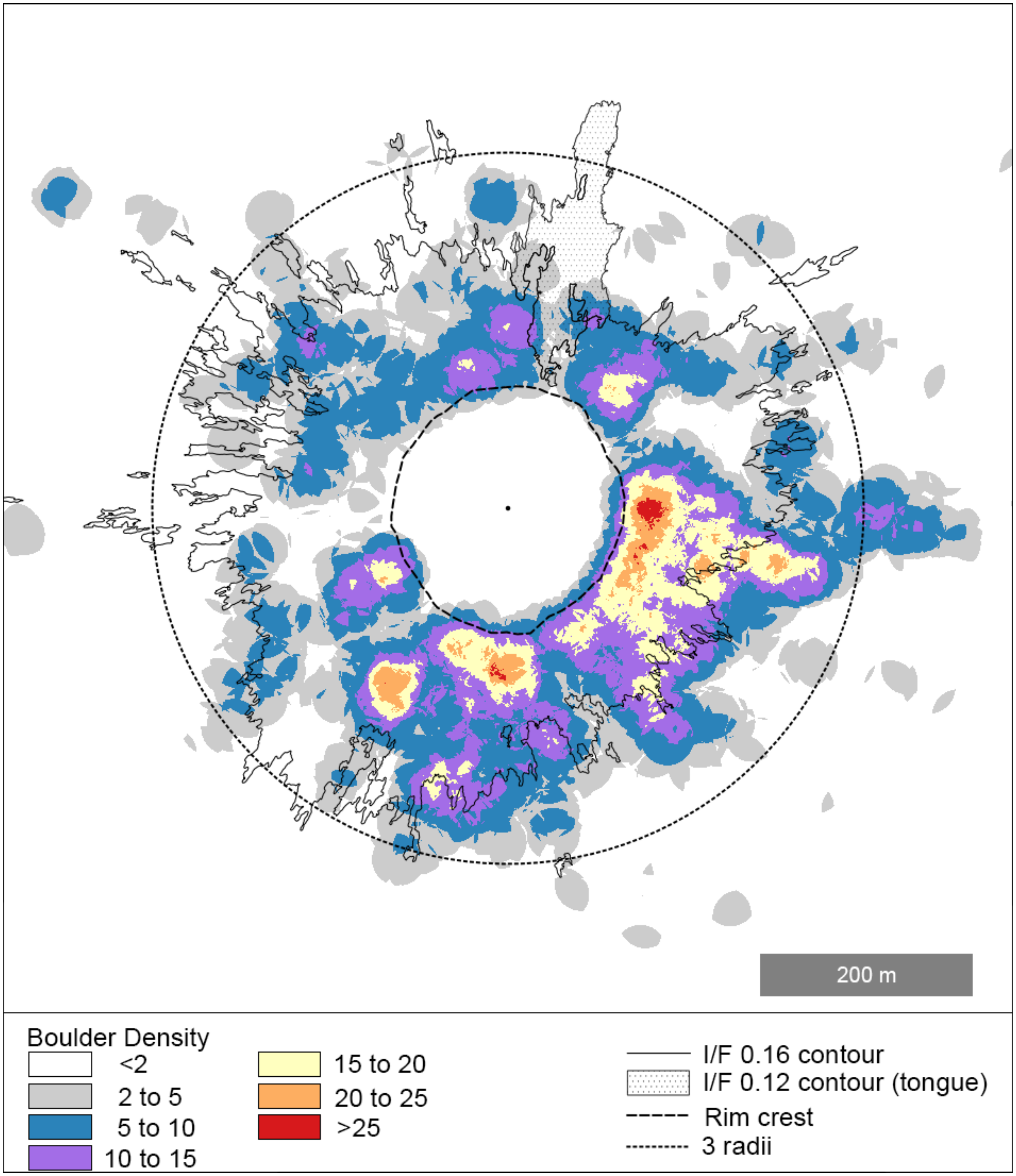


**Supplemental Figure 2. Boulder density map**. Number of boulders (D > 1 m) computed within a 25 m radius. A solid black line indicates the outer edge of the continuous ejecta, and a black stipple pattern locates the northern tongue. The inner large-dash line indicates the crater rim, the outer small-dash line indicates a 3-radii range from the crater center, and the dot indicates the center of the crater. Map area is 950 meters wide, north is up.

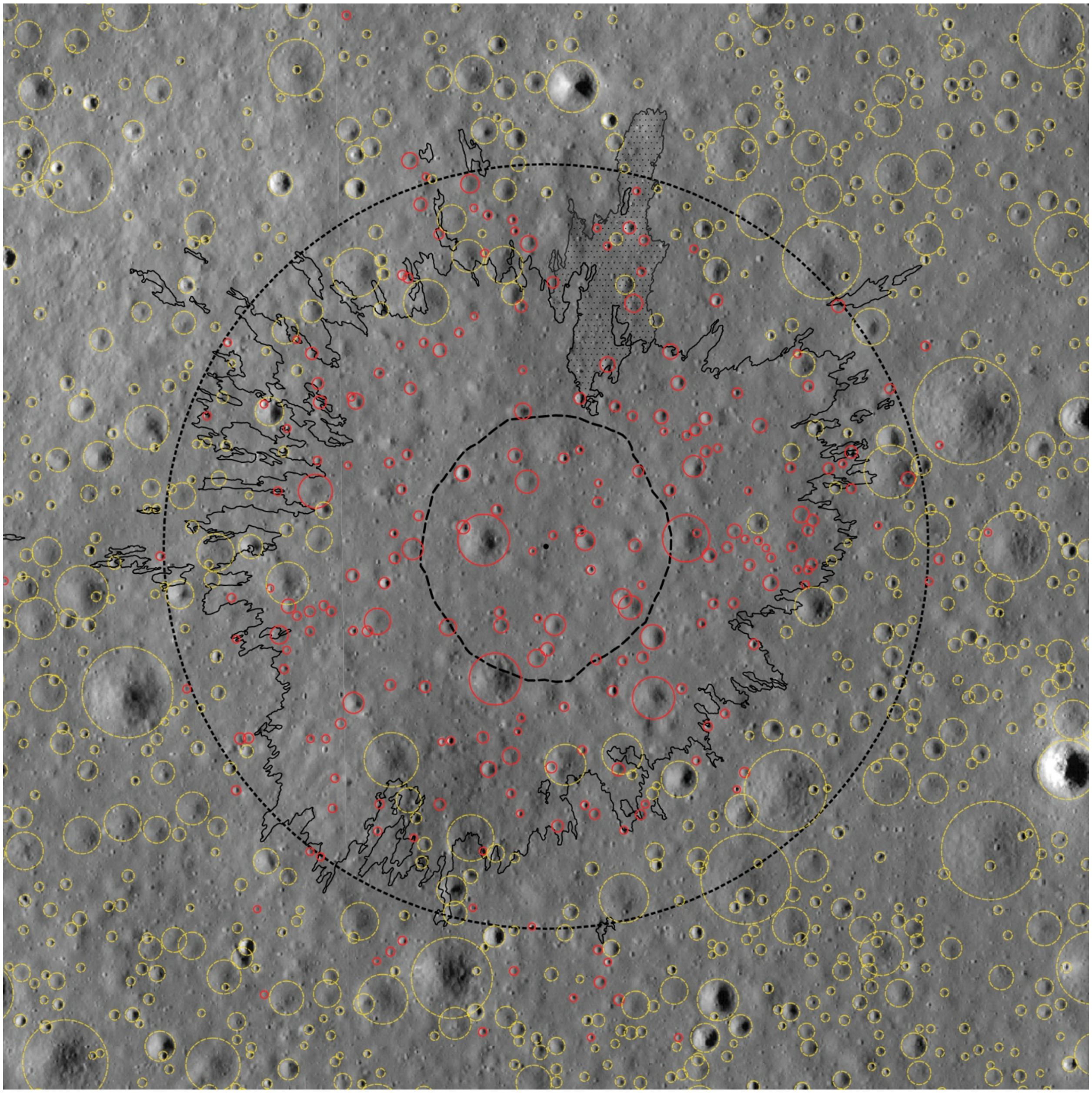

**Supplemental Figure 3. Crater distribution at impact site before the event.** Craters (D > 6 m) with red outlines are unrecognizable after the event; those in yellow are recognizable. Solid black line indicates the outer edge of continuous ejecta, black stipple pattern locates the northern tongue, and a dot indicates the center of the crater the outer small-dash line, indicates a 3-radii range from the crater center. Image width is 950 m, north is up, M146540098L,R.

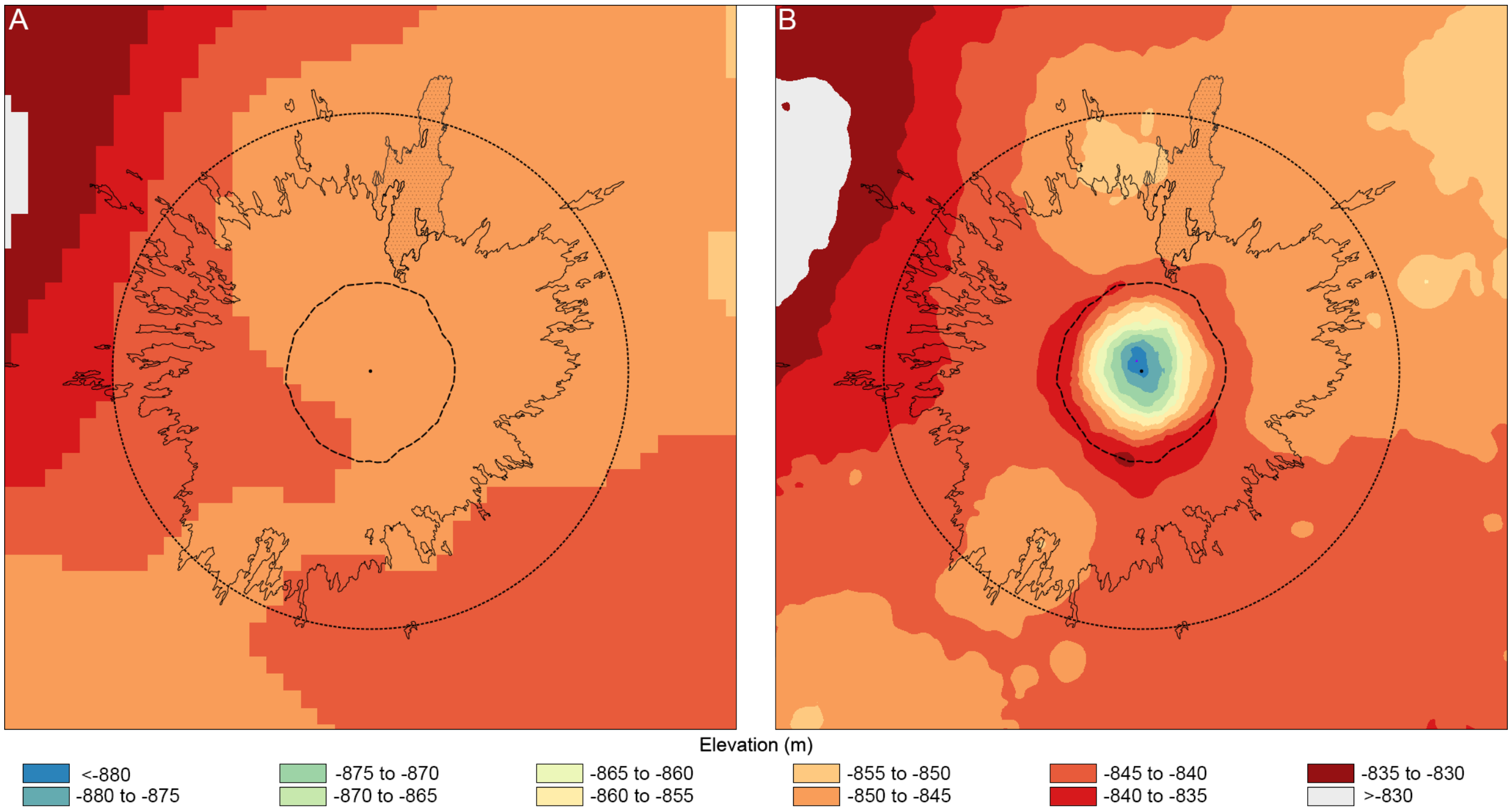


**Supplemental Figure 4. McGetchin crater topography before and after topography.** A) Kaguya stereo-based DTM resampled from original 22-m pixels to match NAC stereo-based topography (B) sampled at 3 meters. Color labels indicate the binned elevations, a black dot indicates the center of the crater, the dotted line is 3-radii from the crater center, and the dashed line is the crater rim. A dot marks the crater center; each panel is 950 meters wide, with north.
